# Cellular and Molecular Mechanisms of Ozone Therapy: Present Knowledge and Prospective Applications

**DOI:** 10.3390/ijms232012586

**Published:** 2022-10-20

**Authors:** Manuela Malatesta, Barbara Cisterna, Manuela Costanzo

**Affiliations:** Department of Neurosciences, Biomedicine and Movement Sciences, University of Verona, I-37134 Verona, Italy

As a complementary, adjuvant or palliative cure, ozone therapy has increasingly been used globally on a wide variety of diseases. The administration of oxygen–ozone (O_2_-O_3_) mixtures has been proven to have therapeutic potential, but the observational evidence still needs to be adequately supported by convincing mechanistic explanations. This Special Issue is aimed at illustrating established and novel knowledge on the cellular and molecular mechanisms accounting for the successful application of ozone therapy to different pathological conditions.

The administration of O_2_-O_3_ mixtures was observed to have immunomodulatory effects in peripheral organs, and to beneficially affect the aging brain. Based on this clue, Michael Bette and co-workers [1] investigated the therapeutic potential of a five-day intraperitoneal treatment with O_2_-O_3_ in a mouse model of amyotrophic lateral sclerosis (ALS), at the beginning of the disease’s symptomatic phase. The authors observed that O_2_-O_3_ had no effect on survival, gross body weight, motor performance, or disease duration; however, neurodegeneration was slowed down and microglia proliferation was less pronounced in the brainstems of O_2_-O_3_-treated ALS mice. In adult ALS mice, the number of monocytes was reduced in the blood, spleen, and mesenteric lymph nodes, but O_2_-O_3_ administration prevented further monocyte decrease at an advanced disease stage. These results are suggestive of a neuroprotective and possibly anti-inflammatory effect of O_2_-O_3_ treatment in ALS mice.

José Baeza-Noci and Rosa Pinto-Bonilla [2] provided a systemic overview of ozone as a potential new chemotherapeutic treatment. Starting with an observation that preclinical studies on cells in vitro and on animals in vivo demonstrated the possibility of ozone effectively inducing cancer cell damage in a harmless way for non-cancer cells, the authors underlined that “few clinical articles have been published, and so, there is small evidence-based support for its clinical use in cancer patients”. A review was thus conducted on a total of 23 articles dealing with in vitro, in vivo, and clinical studies. Baeza-Noci and Pinto-Bonilla concluded that much preclinical investigation is needed whereby more cancer cell lines and different ozone dosages are tested, as it was found that different cancer cell lines are not equally affected by ozone; in addition, more in vivo studies are necessary to validate the systemic use of ozone as a chemotherapeutic agent.

The paper by María de los Ángeles Erario and co-workers [3] aimed to explain the action mechanisms of ozone therapy on extruded disc herniation, which occurs when the nucleus pulposus squeezes through a weakness or tear in the annulus. The host immune system perceives the extruded nucleus material as a non-self, so that an immune response and inflammation are triggered. In the authors’ view, ozone therapy modulates this immune response through the activation of macrophages, facilitating the passage from their M1 to M2; phagocytosis of the extruded material occurs, with passage from an inflammatory to a reparative phase. 

Renate Viebahn-Haensler and Olga Sonia León Fernández [4] illustrated and discussed the effect and mechanisms of low doses of ozone in chronic inflammation: here, ozone acts as a bioregulator in diseases that are biochemically characterized by high oxidative stress. Ozone at low doses induces moderate oxidative stress and represents an effective hormetic strategy. International guidelines have been established to select the proper materials and the effective concentration and dosage ranges to be used, in an attempt to obtain the best therapeutic benefit. In the treatment of chronic inflammatory diseases, clinicians should integrate the administration of medical ozone with conventional therapeutics and drugs; this would allow the achievement of a synergistic effect thanks to ozone bioregulatory mechanisms and reduce drug side effects, particularly on the liver and kidney.

Barbara Cisterna and co-workers [5] focused on the effects of low O_2_-O_3_ concentrations on fibroblasts—cells ubiquitously located in the connective tissues, where they play structural and functional roles in the body architecture and the homeostasis of tissue-resident cells—in health and disease. The authors used an established human fibroblast cell line in vitro, and combined light and electron microscopy, Western blotting, real-time quantitative polymerase chain reaction, and multiplex assays for cytokines, in a multimodal approach aimed at exploring a panel of cell structural and functional features. Multiple effects were induced in fibroblasts following the administration of O_2_-O_3_ gas mixtures: in non-activated fibroblasts, the exposure to O_2_-O_3_ increased proliferation, the formation of cell surface protrusions, the antioxidant response, and the secretion of interleukin-6 and TGF-β1, whereas in LPS-activated fibroblasts, only the antioxidant response and cytokine secretion were stimulated. These results allowed the authors to conclude that O_2_-O_3_ at low concentrations is able to stimulate non-activated fibroblasts towards an activation-like response, whereas in LPS-activated fibroblasts, the cells’ protective reaction is improved.

The articles in this Special Issue provide interesting and novel evidence on the effects and mechanisms of ozone on cells and tissues; however, the authors all agree that intense additional work is warranted in both preclinical research and clinical trials, to make O_2_-O_3_ administration a fully and unanimously accepted approach in clinical practice.
